# Metabolic Disturbances Associated with Systemic Lupus Erythematosus

**DOI:** 10.1371/journal.pone.0037210

**Published:** 2012-06-19

**Authors:** Tianfu Wu, Chun Xie, Jie Han, Yujin Ye, Jim Weiel, Quan Li, Irene Blanco, Chul Ahn, Nancy Olsen, Chaim Putterman, Ramesh Saxena, Chandra Mohan

**Affiliations:** 1 University of Texas Southwestern Medical Center, Dallas, Texas, United States of America; 2 Metabolon Inc, Research Triangle Park, North Carolina, United States of America; 3 Albert Einstein College of Medicine, New York, New York, United States of America; Institut Pluridisciplinaire Hubert Curien, France

## Abstract

The metabolic disturbances that underlie systemic lupus erythematosus are currently unknown. A metabolomic study was executed, comparing the sera of 20 SLE patients against that of healthy controls, using LC/MS and GC/MS platforms. Validation of key differences was performed using an independent cohort of 38 SLE patients and orthogonal assays. SLE sera showed evidence of profoundly dampened glycolysis, Krebs cycle, fatty acid β oxidation and amino acid metabolism, alluding to reduced energy biogenesis from all sources. Whereas long-chain fatty acids, including the n3 and n6 essential fatty acids, were significantly reduced, medium chain fatty acids and serum free fatty acids were elevated. The SLE metabolome exhibited profound lipid peroxidation, reflective of oxidative damage. Deficiencies were noted in the cellular anti-oxidant, glutathione, and all methyl group donors, including cysteine, methionine, and choline, as well as phosphocholines. The best discriminators of SLE included elevated lipid peroxidation products, MDA, gamma-glutamyl peptides, GGT, leukotriene B4 and 5-HETE. Importantly, similar elevations were not observed in another chronic inflammatory autoimmune disease, rheumatoid arthritis. To sum, comprehensive profiling of the SLE metabolome reveals evidence of heightened oxidative stress, inflammation, reduced energy generation, altered lipid profiles and a pro-thrombotic state. Resetting the SLE metabolome, either by targeting selected molecules or by supplementing the diet with essential fatty acids, vitamins and methyl group donors offers novel opportunities for disease modulation in this disabling systemic autoimmune ailment.

## Introduction

SLE is a systemic autoimmune disease resulting in chronic activation of self-reactive lymphocytes and pro-inflammatory myeloid cells, and inflammation targeting multiple end organs including the kidneys, brain, joints and skin. The molecular basis for the various manifestations of this autoimmune disease and the impact of the systemic autoimmune process on basic metabolic processes in the body are currently obscure. In addition, the currently available yardsticks to diagnose and prognosticate the disease are far from optimal.

In search for novel insights on the disease, as well as potential disease markers, serum samples from SLE patients were subjected to a comprehensive metabolic scan using LC/MS and GC/MS based platforms, and a library of >2000 metabolite standards. Statistically significant differences were noted in >100 metabolites, falling into several metabolic pathways. The primary metabolic scan and subsequent validation assays using an independent cohort of subjects reveal metabolic imbalances in multiple processes, including glycolysis, the Krebs cycle, fatty acid (FA) β-oxidation, lipid biosynthesis, eicosanoid biosynthesis, and methyl group metabolism. The lupus metabolome was also marked by elevated oxidative stress, insufficient substrates for energy biosynthesis, imbalanced lipid profiles, and elevated inflammatory markers. In addition to providing novel insights on the metabolic fabric underlying SLE, these studies also point to potential disease markers, therapeutic targets, and imbalances that may be amenable to dietary correction.

## Results

**Figure 1:**
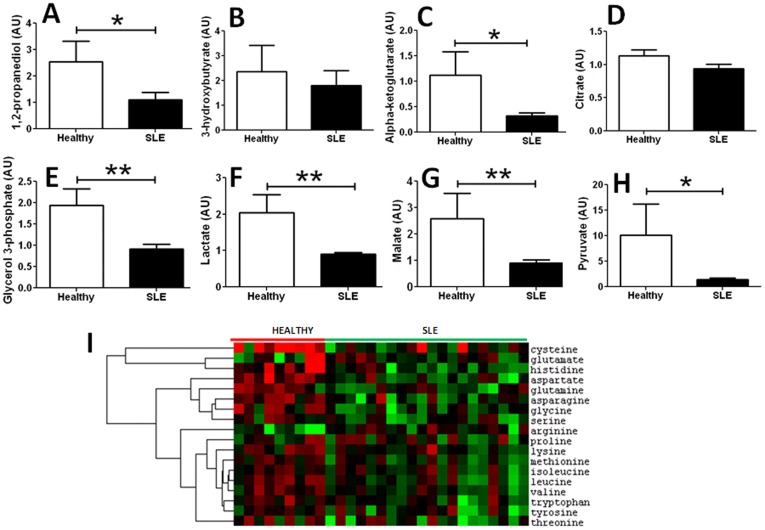
Key metabolic imbalances in SLE affecting carbohydrate, lipid or amino acid metabolism. The sera of 20 SLE patients and 9 healthy controls were comprehensively scanned for differences in small molecules using LC/MS and GC/MS platforms, referred to as the “metabolomic scan”. Shown are the mean metabolite levels of 3 glycolytic intermediates (A–C), three Kreb’s cycle intermediates (D–F), and two products of fatty acid β-oxidation (G–H). Open bars = healthy controls; closed bars = SLE patients. (*,P<0.05; **,P<0.01; ***,P<0.001). Plotted in (I) is a heatmap of serum amino acid levels in healthy subjects (first 9 columns) versus SLE patients (rightmost 20 columns), as determined by the metabolomic scan described above. Red = elevated; green = reduced, relative to the mean levels of the metabolite within the 29 study subjects. The actual mean levels of the metabolites are listed in Supplementary Table S1.

For the primary metabolomic scan, serum from 20 SLE patients (whose particulars are summarized in Table 1) was compared to serum metabolities of 9 healthy controls, using a combined LC/MS and GC/MS based approach, and a library of >2000 metabolite standards. In comparing the metabolites in SLE sera and healthy controls, >100 metabolites were significantly different in SLE, as tabulated in Supplementary Table S1. Reference to the Kyoto Encyclopedia of Genes and Genomes (KEGG, release 41.1, http://www.genome.jp/kegg) helped identify the metabolic pathways that the dysregulated metabolites belonged to. A substantial fraction of the observed differences pertained to energy metabolism. Energy from carbohydrates can be derived through glycolysis. This potential energy source was evidently reduced in SLE, extrapolating from the significant reduction in key intermediates in this pathway including glycerol-3 phosphate, pyruvate and lactate (Fig. 1A–1C). Even more energy can be derived via the Krebs cycle; however, this was also significantly dampened in SLE patients, as marked by the reduced serum levels of malate, citrate and α-ketoglutarate in SLE sera (Fig. 1D–1F). Energy derivation from lipids was also evidently reduced in SLE, based on the significantly reduced levels of intermediates of β-oxidation, 1,2 propanediol and 3-hydroxybutyrate (BHBA) (Fig. 1G–1H, and Supplementary Table S1). In the absence of energy derivation from carbohydrates and lipids, amino acids could emerge as potential energy sources. However, all ketogenic and glucogenic amino acids (with the exception of arginine) were also significantly dampened in SLE (Fig. 1I; Supplementary Table S1).

**Table 1:** Demographics and Clinical characteristics of SLE patients used for the metabolomic profiling.

No.	20
Female, no.	15
Age, median, years (range)	33.8 (18–40)
Race: African American/Hispanic, no.	9/10
BMI, kg/M^2^, median (interquartile)	29.9 (22.5–36.7)
SLEDAI, median (range)	5 (0–18)
Protein:creatinine ratio, mg/mg, mean	2.388
Serum Cr, mg/dl, mean	1.22
Positive anti-dsDNA, no.	8
Hypocomplementemia, no. (total no.tested)	9 (19)
Comorbidities	
Diabetes Melitus	1
Hypertension	14
Dyslipidemia	4
Deep venous thromboembolism	4
Cardiovascular disease	1
Others	7
Current medications, no.	
Prednisone	15
Mycophenolic acid	8
Azathioprine/MTX	3
Cyclophosphamide	2
Angiotensin blocking agents	7
Hydroxychloroquine	9

**Figure 2:**
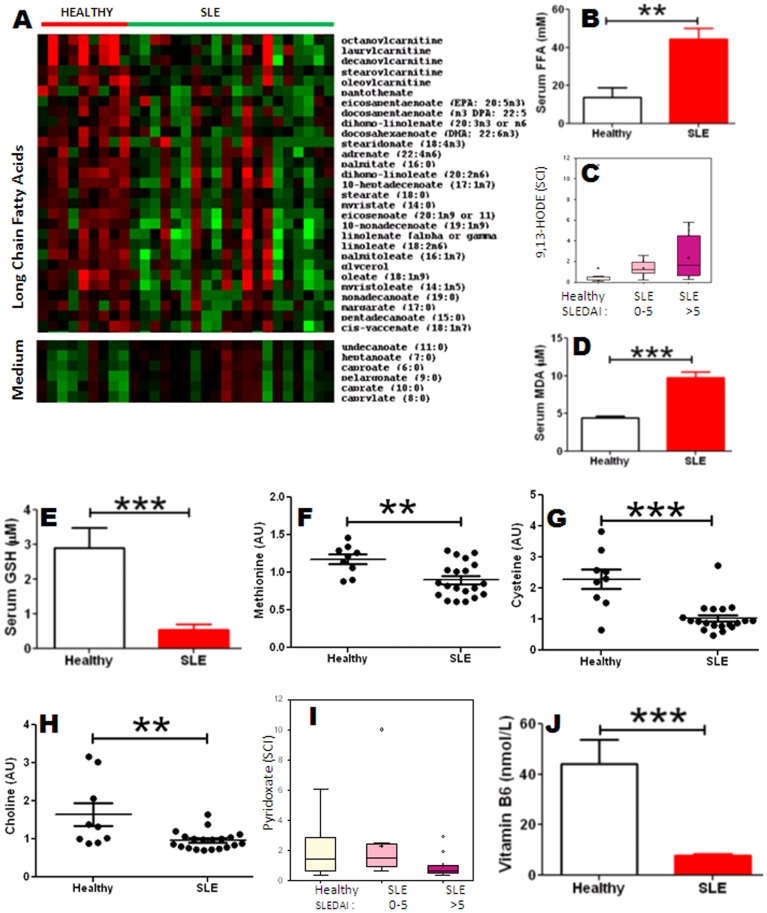
Lipid profiles and methyl group donors in SLE. Plotted in the heatmap in (A) are the serum levels of long chain fatty acids (FA) and medium chain FA in 9 healthy controls and 20 SLE subjects, as determined by the metabolomic scan. Presentation details are as in Fig. 1(I). In the metabolomic scan, additional differences were noted in the serum levels of 9-HODE and 13-HODE (C), methionine (F), cysteine (G), choline (H) and vitamin B6 (I); presentation details are as in Fig. 1. In (C) and (I), the SLE patients have been segregated into 2 groups - mild SLE (SLEDAI <6; N = 10) and active SLE (SLEDAI >5; N = 10). Also plotted are validation assays for serum levels of free fatty acids (FFA; B), the lipid peroxidation marker, MDA (D), glutathione (GSH; E), and vitamin B6 (J), ascertained in an independent cohort of 38 SLE patients and 14 healthy controls, using commercially available assays, independent of the original metabolomic scan. Each dot represents data from an individual subject (*,P<0.05; **,P<0.01; ***,P<0.001).

Another cluster of molecules that were significantly reduced in SLE included all long chain FA and acyl-carnitines, which are required for ferrying FA into the mitochondria for β-oxidation and energy release, as captioned in the heatmap in Fig. 2A. Interestingly, all essential FA (which are FA that cannot be synthesized by the body, but are of dietary origin) were also significantly reduced in SLE. These included the polyunsaturated fatty acids (PUFA), both n3-PUFA (α-linolenic acid, eicosapentaenoate or EPA, docosahexaenoate or DHA) as well as n6-PUFA (linoleic acid, γ-linolenic acid, dihomo γ-linolenate or DGLA, and dihomolinoleate) (Fig. 2A; Supplementary Table S1). In contrast to the reduction in long-chain FA, SLE sera exhibited elevated levels of medium chain FA (Fig. 2A). An independent assay using an orthogonal platform confirmed the significantly elevated levels of free FA (FFA), in an independent cohort of SLE patients (Fig. 2B). Collectively, these studies indicate that free FA of medium chain length are elevated in SLE, while long chain FA and essential PUFA were reduced.

SLE sera exhibited a profound degree of lipid peroxidation, as marked by the elevated levels of the products, 9-HODE and 13-HODE, with these elevations being even more pronounced in patients with active disease (Fig. 2C). These observations were validated using an independent platform and independent serum samples (Fig. 2D). These findings point to an increased level of oxidative stress in SLE. Concordant with these findings, the levels of the leading intracellular anti-oxidant, glutathione (GSH) was significantly reduced in SLE (Fig. 2E).

Methyl group donors, which are necessary for the regeneration of glutathione, were all significantly reduced in SLE, including methionine, cysteine, and choline (Fig. 2F–H). The reduced choline levels were, as one would predict, accompanied by significant reductions in phosphocholine levels (Supplementary Figure S1). Besides methyl group donors, the regeneration of glutathione requires various co-factors, including vitamin B6; this vitamin was significantly reduced in SLE sera, with the degree of reduction correlating with disease activity (Fig. 2I) This was validated using an independent cohort of SLE sera (Fig. 2J). Vitamin B5 and alpha-tocopherol were also reduced in SLE sera (Supplementary Table S1).

**Figure 3:**
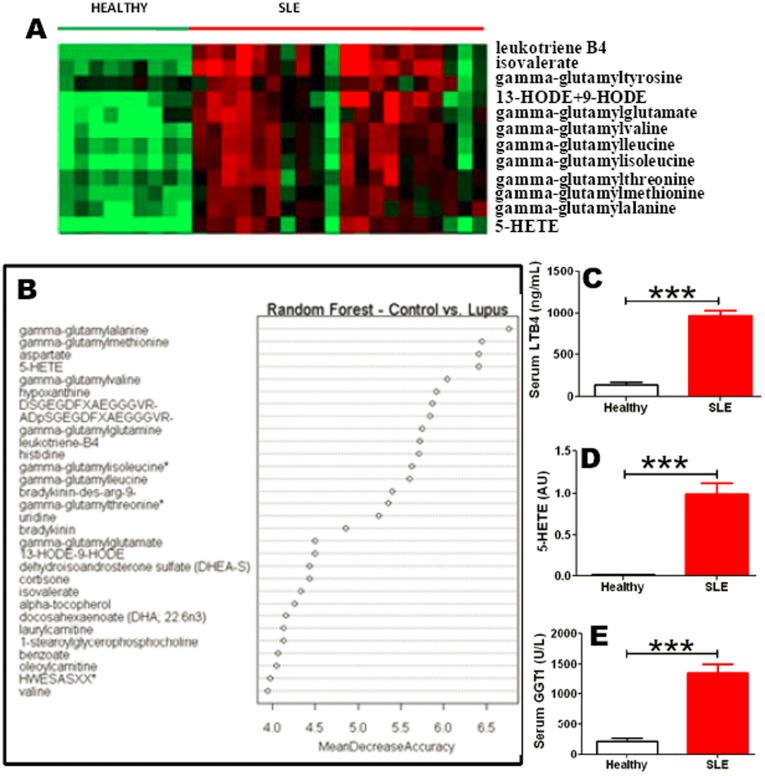
Metabolic markers that best distinguish SLE from healthy controls. Plotted in (A) is a heatmap of a cluster of metabolites that were elevated in SLE sera; presentation details are listed in Fig. 1(I). The metabolites that were best at discriminating SLE from controls (based on the results from the original metabolomic scan of 20 SLE patients and 9 healthy controls) were identified and ordered using a Random Forest analysis algorithm (B). The markers are listed in decreasing order of disease-discriminatory potential. Also plotted are validation assays for serum levels of leukotriene B4 (C), 5-HETE (D), and serum GGT1 (E) ascertained in an independent cohort of 38 SLE patients and 14 healthy controls, using commercially available kits, independent of the original metabolomic scan (*,P<0.05; **,P<0.01; ***,P<0.001).

In contrast to the reduced levels of most metabolites, a couple of metabolites were significantly elevated in SLE sera, as summarized in the heatmap in Fig. 3A. Besides the increase in lipid peroxidation products (9-HODE and 13-HODE), several gamma-glutamyl peptides, and two eicosanoid metabolites in the n6-PUFA pathway, Leukotriene B4 (LTB4) and 5-HETE were also significantly elevated in SLE. Elevations were also noted in the serum levels of fibrin-degradation peptides and bradykinin in SLE (Supplementary Table S1). A Random Forest analysis was performed to identify the metabolites that had the greatest discriminatory potential to distinguish SLE from controls. The best discriminators included serum levels of gamma-glutamyl peptides, 5-HETE, leukotriene B4, bradykinins, fibrin degradation products and lipid peroxidation products (Fig. 3B). The increases in LTB4 and 5-HETE were validated using independent assay platforms and an independent cohort of SLE patients (Fig. 3C–D). As noted above, almost all gamma-glutamyl peptides were also significantly elevated in SLE (Fig. 3A). These peptides not only indicate a vigorous attempt to generate glutathione, they also suggest that the responsible enzyme, gamma-glutamyl transaminase, GGT, may be more active or elevated in SLE. This prediction was confirmed using an independent assay and an independent cohort of SLE patients (Fig. 3E).

**Figure 4:**
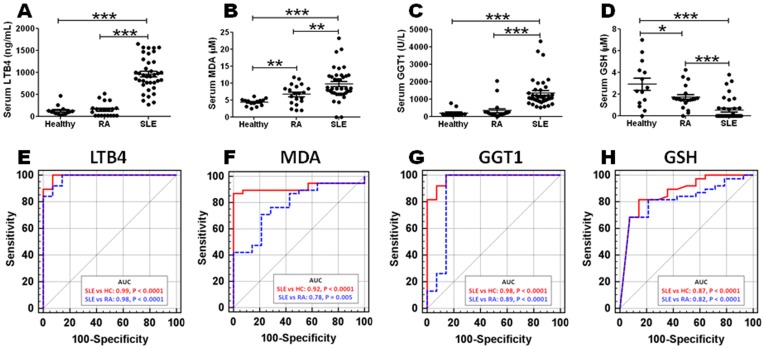
Sensitivity and specificity profiles of new metabolic markers in SLE. The levels of the 4 markers indicated were tested in serum samples from RA patients (open dots; N = 20) and SLE patients (closed dots; N = 38) (A–D). Each dot represents data from a single individual. (*,P<0.05; **,P<0.01; ***,P<0.001). The dotted line represents the mean serum levels in healthy controls (N = 14). The potential of the different markers in distinguishing SLE from healthy controls (red bold line), or from RA disease controls (black dotted line) were also analyzed using ROC curves, as displayed for leukotriene B4 (E), MDA (F), GGT1 (G) and glutathione (H), based on the serum levels observed in the independent cohort of 38 SLE patients, 20 RA patients and 14 healthy controls, in the validation assays plotted in A–D. The ROC curves plot (1-Specificity) % on the x-axis versus the Sensitivity (%) on the Y-axis, for each marker. AUC = Area under ROC curve.

To gauge the disease specificity of these metabolic markers, they were next examined in another pro-inflammatory, chronic, systemic autoimmune disease, rheumatoid arthritis (RA). The alterations in serum leukotriene B4, MDA, GGT, and glutathione noted in sera from RA patients were not as profound as those observed in SLE sera (Fig. 4A–D). Importantly, receiver operator curves (ROC) underscored the excellent predictive values of serum leukotriene B4 (AUC = 0.99), lipid peroxidation marker MDA (AUC = 0.92), GGT1 (AUC = 0.97), and glutathione (AUC = 0.84) in distinguishing SLE from healthy controls (Fig. 4E–H; redlined). Indeed, all 4 markers were also very effective in distinguishing SLE from RA, as revealed by the respective (black, dotted) ROC curves in Fig. 4E–H. Interestingly, RA patients exhibited modest elevations in serum MDA and reduction in glutathione compared to healthy controls, though not as pronounced as those seen in SLE (Fig. 4B, 4D).

A couple of metabolite levels in SLE sera showed significant association with some of the medications the patients were on, including valine, urate, phenylalanine, leucine, citrulline and methyl butryl carnitine with prednisone, acetaminophen sulfate and acetamidophenylglucoronide with lisinopril, and guanosine, theophylline and tryptophan betaine with mycophenolic acid, as listed in Supplementary Table S1. Besides these, the other metabolic changes described in this report showed no association with the nature of the medications the patients were on. We have re-evaluated this analysis with the validation data sets, and have confirmed that the serum levels of MDA, GSH, Leukotriene B4 and GGT1 in SLE patients showed no significant association or correlation with corticosteroid, plaquenil or MMF therapy, even when the dosages were factored in.

## Discussion

**Figure 5:**
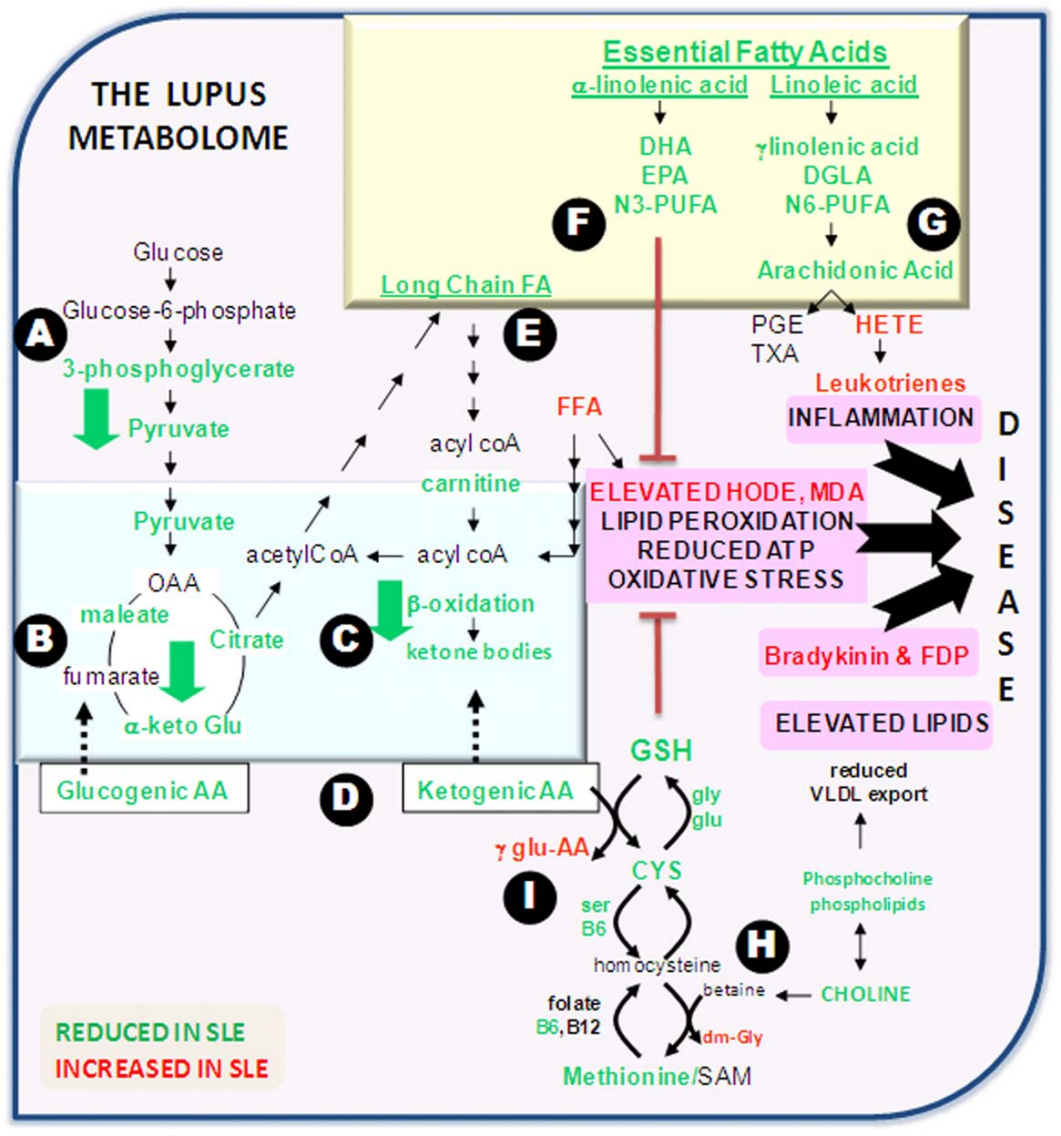
An overview of the metabolic imbalances in SLE. The most significant metabolic alterations in SLE have been organized into different biochemical pathways, including glycolysis (A), Krebs cycle (B), fatty acid oxidation (C), amino acid pools (D), lipid biosynthesis (E), essential FA (F), eicosanoid biosynthesis (G) and methyl group interchange pathways (H) leading to glutathione generation (I). Metabolites that were elevated in SLE are in red font, while reduced metabolites are in green font. Mitochondrial events are blue-boxed, while events that take place in the endoplasmic reticulum are yellow-boxed. Salient metabolic consequences that can potentially contribute to the manifestations of SLE are highlighted in pink.

This first comprehensive profiling of the metabolomic landscape in systemic lupus erythematosus reveals a wide array of disturbances, as summarized in Figure 5. First and foremost is the profound degree of shutdown of all energy generating pathways, including glycolysis (Fig. 5A), Krebs cycle (Fig. 5B), β-oxidation of lipids (Fig. 5C), and the pool of available amino acids (Fig. 5D). Assuming these changes are reflective of intracellular changes within these patients, these findings point to a drastic reduction of ATP generation, and are consistent with earlier reports indicating that SLE T-cells are less efficient at ATP generation [1]. We postulate that this may in part represent the molecular basis for the chronic fatigue that SLE patients typically experience, though this needs to be formally proven.

Several imbalances were also noted in the patients’ lipid profiles. Whereas all long chain FA were reduced (Fig. 2, 5E), medium chain FA were elevated. Whereas the former are primarily synthesized de novo, the latter are often of dietary origin, since they are better absorbed. The reduced long chain FA may be due in part to the reduced availability of the component building blocks - acyl coA, as well as available co-factors. In addition, the raised free fatty acids and medium chain FA levels, coupled with the reduction in essential FA (both n3-PUFA and n6-PUFA; Fig. 5F) may point to a diet that is high in saturated:unsaturated ratios. Other than dietary differences, the significant reduction in essential FA could also be due to accelerated metabolic consumption or conversion, either through lipid peroxidation or heightened leukotriene synthesis (Fig. 5G). Whereas n3-PUFAs are significantly reduced (and possibly peroxidized), the downstream products of the n6-PUFA pathway, namely 5-HETE and leukotriene B4 are significantly elevated in SLE. Dampened n3-PUFA:n6-PUFA ratios may engender a pro-inflammatory milieu, as reported by others [2], [3]. Collectively, the observed lipid imbalances, pro-inflammatory milieu and the pro-thrombotic state in SLE resonate well with the increased cardiovascular complications documented in SLE [4], [5], [6].

The profound degree of lipid peroxidation seen in SLE is likely to be a readout of increased oxidative damage due to the generation of free radicals in the mitochondria. Indeed, increased oxidative stress and the reduced availability of anti-oxidants in SLE have been documented in previous reports [7], [8]. Our present findings confirm the reduced levels of the key intracellular antioxidant, glutathione (GSH) in SLE (Fig. 2, 5I). This reduction in GSH is likely to be the end-result of reduced generation and/or increased consumption. The generation of GSH and its intermediates requires component amino acids, methyl group donors, and various cofactors, all of which are profoundly reduced in SLE (Fig. 1I, 2F–J). The reduction in methyl group donors (e.g., methionine, choline, cysteine; Fig. 5H) is also likely to curtail other molecular mechanisms that are dependent upon methylation, including DNA and histone methylation, lipid metabolism, and the synthesis of various mediators. Indeed, global DNA hypomethylation, accompanied by the activation of various genes has been documented in human SLE [9], [10], and this may in part be attributed to the drastic reduction in available methyl group donors in these patients. There are however indications that the system is vigorously attempting to generate GSH, based on the striking elevations in various gamma-glutamyl peptides, as well as dimethyl glycine, both of which are by-product footprints of these metabolic cascades (Fig. 3A, 5I; Supplementary Table S1).

The reduced levels of choline are, as expected, accompanied by reduced levels of phosphocholines (Fig. 5H). Since phosphocholines constitute integral membrane components, this deficiency could adversely impact membrane structure/dynamics, as well as cell signaling and function in SLE patients – predictions that await formal testing. Furthermore, reduced phosphocholine will impair the export of lipids as VLDL out of the liver. In the face of elevated FFA, these changes could pave the way towards hepatic steatosis and fatty liver, and their associated co-morbidities. Whether SLE patients harbor these hepatic alterations warrants systematic study.

Presently, it is unclear if the serum metabolic changes observed in this study arise from free metabolites in the sera or from cell-derived particles (e.g., cellular debris, exosomes, etc). Irrespective, these disturbances are likely to be reflective of the intracellular metabolic alterations in these patients, arising from leukocytes or non-immune cells (e.g., endothelial cells, hepatocytes, etc). Though the cascades diagramed in Fig. 5 might have arisen from multiple independent triggers, it is tempting to speculate that subsets of these alterations may be interconnected to each other, and to immune activation. For instance, robust and repeated activation of autoreactive lymphocytes (by autoantigens), various myeloid cells and other non-immune cells may be the wellspring of redox radicals. This in turn could result in profound oxidative damage (including lipid peroxidation) and consumption of GSH. Refocusing all of the cell’s efforts towards GSH regeneration could have rippling effects on the cellular stores of amino acids, methyl groups and various co-factors, the depletion of which may dysregulate additional interlinked metabolic pathways. Whether genetic and/or epigenetic factors might also directly regulate any of the cascades depicted in Fig. 5 is an open question. Whereas some of the observed changes may be the consequence of disease, others may well play a role in causing or aggravating various manifestations of the disease, as implied in Fig. 5 (pink boxes).

These metabolic alterations have potential diagnostic and therapeutic implications. Based on the validation assays using independent serum samples and independent assay platforms, the serum levels of leukotriene B4, the lipid peroxidation marker MDA, gamma-glutamyl peptides or GGT, and glutathione emerge as excellent predictors of SLE, with superior specificity and sensitivity profiles (Fig. 4), effectively differentiating SLE from healthy controls as well as another pro-inflammatory, chronic systemic autoimmune disease, RA. Moreover, the SLE-associated metabolic changes reported here are distinct from those associated with other diseases whose metabolomes have been comprehensively profiled, including other autoimmune diseases [11], [12] and cancer [13]. Although all four of the markers displayed in Fig. 4 were significantly altered in SLE sera, they did not correlate significantly with SLEDAI, anti-dsDNA titers, renal disease or disease duration (data not shown). Interestingly, although the peroxidation marker MDA did not correlate with disease activity in SLE, other lipid peroxidation markers such as 9/13-HODE did show higher associations with higher disease activity (Fig. 2C); the significance of these subtle differences currently remain unknown. Interestingly, the increased serum levels of leukotriene B4 and reduced serum levels of glutathione correlated with age, alluding to the importance of factoring in age differences when examining these two markers.

Finally, the metabolic landscape in SLE may lend itself to disease modulation by targeted therapeutics and/or dietary means. The reduced levels of n3-PUFA (which is totally diet-derived), could perhaps be corrected by dietary supplementation particularly since fish oil supplements have been shown to be effective in murine lupus [14], and in limited clinical trials in SLE patients [15]. The reduced levels of various co-factors call for adequate vitamin supplementation (in particular, the B vitamins) as adjunctive therapy in SLE. Since dietary choline is the major source of methyl groups, supplementing the diet with choline and its precursors (notably, lecithin) may also be important. Whether dietary anti-oxidants can alter the intra-cellular redox balance in SLE remains to be established. Regardless, resetting the imbalanced metabolome offers new and exciting opportunities for disease modulation in SLE.

## Materials and Methods

### Patient Samples

For the primary metabolomic scan, all 20 SLE patients were drawn from the Renal Clinic at UT Southwestern Medical Center, Dallas, TX, of whom 10 had active renal disease with SLEDAI >5. Patients were recruited as and when they were seen for their routine outpatient clinic visits, as long as they met all recruitment criteria. The patients’ characteristics and the medications they were on are detailed in Table 1. The mean body mass index (BMI) of the SLE patients (29.9) was not significantly different from that of the general population in Dallas (mean BMI = 29; N = 6101), from which all healthy controls were drawn [16]. Healthy control individuals in the Dallas Autoimmune Disease Registry [17] were recruited from the Dallas area and screened to be sure that the donor did not have autoimmune disease personally or in first-degree relatives; 78% of the controls were African American or Hispanic females. The mean BMI of the healthy controls used in this study was 26.9, not significantly different from that of the SLE patients studied (P>0.10), while their mean age was 36. For all validation assays, an independent cohort of SLE patients (N = 38; average age = 38; average BMI = 28.7; average SLEDAI = 7) was used, drawn from the rheumatology clinic at Albert Einstein College of Medicine, New York. More than 80% of these subjects were either of African-American or Hispanic origin. Disease activity was gauged using SLEDAI (SLE disease activity index) [18]. All Rheumatoid Arthritis (RA) patients were also drawn from the Albert Einstein College of Medicine, New York, and were age, gender and ethnicity matched to the SLE patients.

All human research was approved by the UT Southwestern institutional review board (IRB). All research was conducted with informed written consent, and all clinical investigation were conducted according to the principles expressed in the Declaration of Helsinki.

### Metabolomic Profiling - Sample Preparation

The metabolomic profiling procedures have been detailed previously [19]. Human sera were obtained from healthy controls (N = 9) and SLE patients (N = 20). Serum samples were collected using BD vacutainer serum tubes (Ref 367820), spun at 1500 RCF for 10 minutes at room temperature. The retrieved serum was then aliquoted and stored in −80. For the metabolomic analysis, frozen serum aliquots were thawed, and processed using an automated MicroLab STAR® system (Hamilton Company), using a series of organic and aqueous extractions to remove the protein fraction in sera while allowing maximum recovery of small molecules. Recovery standards were added prior to the first step in the extraction process for QC purposes. The resulting extract was divided into two fractions; one for analysis by LC and one for analysis by GC. Samples were placed briefly on a TurboVap® (Zymark) to remove the organic solvent, and then frozen and dried under vacuum. Samples were then prepared for the appropriate instrument, either LC/MS or GC/MS.

### Liquid Chromatography/Mass Spectrometry (LC/MS, LC/MS^2^)

The LC/MS portion of the platform was based on a Waters ACQUITY UPLC and a Thermo-Finnigan LTQ-FT mass spectrometer, which had a linear ion-trap (LIT) front end and a Fourier transform ion cyclotron resonance (FT-ICR) mass spectrometer backend. The sample extract was split into two aliquots, dried, then reconstituted in acidic or basic LC-compatible solvents, each of which contained 11 or more injection standards at fixed concentrations. One aliquot was analyzed using acidic positive ion optimized conditions and the other using basic negative ion optimized conditions in two independent injections using separate dedicated columns. Extracts reconstituted in acidic solvents were gradient eluted using water and methanol both containing 0.1% formic acid, while the basic extracts, which also used water/methanol, contained 6.5 mM ammonium bicarbonate. The MS analysis alternated between MS and data-dependent MS^2^ scans using dynamic exclusion. For ions with counts greater than 2 million, an accurate mass measurement could be performed. Accurate mass measurements could be made on the parent ion as well as fragments. The typical mass error was less than 5 ppm. Fragmentation spectra (MS/MS) were typically generated in data dependent manner, while targeted MS/MS was employed in the case of lower level signals.

### Gas Chromatography/Mass Spectrometry (GC/MS)

The samples destined for GC/MS analysis were re-dried under vacuum desiccation for a minimum of 24 hours prior to being derivatized under dried nitrogen using bistrimethyl-silyl-triflouroacetamide (BSTFA). A 5% phenyl-based GC column was used, with the temperature being ramped from 40° to 300°C over a 16 minute period. Samples were analyzed on a Thermo-Finnigan Trace DSQ fast-scanning single-quadrupole mass spectrometer using electron impact ionization. The instrument was tuned and calibrated for mass resolution and mass accuracy on a daily basis.

### Compound Identification

Identification of known chemical entities was based on comparison to a metabolomic library with >2000 entries of purified standards. The combination of chromatographic properties and mass spectra gave an indication of a match to the specific compound or an isobaric entity in the library. Additional entities could be identified by virtue of their recurrent chromatographic and mass spectral nature. These compounds have the potential of being identified by future acquisition of matching purified standards or by classical structural analysis.

### Validation Assays

All validation studies were performed using orthogonal methods other than GC/MS or LC/MS. Glutathione (GSH) was measured using a Glutathione Assay kit purchased from Cayman Chemical, Ann Arbor, MI. Serum MDA was determined using a TBARS Assay kit purchased from Cayman Chemical, Ann Arbor, MI. Free Fatty Acids (FFA) were measured using a Free Fatty Acid Quantification kit purchased from Abcam, Cambridge, MA. Serum GGT1 was measured using a Human Gamma Glutamyltransferase 1 (GGT1) ELISA kit purchased from USCN Life Sciences InC., Wuhan, China. Serum vitamin B6 was assayed by AntiCancer, Inc., San Diego, CA. All assays were performed following the manufacturer’s instructions.

### Statistical Calculation

For pair-wise comparisons, Welch’s t-tests and/or Wilcoxon’s rank sum tests were used. For classification of the best discriminators, Random Forest analysis was used. Random Forests give an estimate of how well we can classify individuals in a new data set into different study groups (e.g., SLE versus healthy controls) [20] in contrast to a t-test, which tests whether the unknown means for two populations are different or not. Random Forests create a set of classification trees based on continual sampling of the experimental units and compounds. Then each observation is classified based on the majority votes from all the classification trees [5]. Statistical analyses were performed using “R” from the Free Software Foundation, Inc. (http://cran.r-project.org/) or Array Studio (Omicsoft, Inc.).

## Supporting Information

Figure S1 **Serum phosphocholine levels in SLE.** Plotted are the serum levels of phosphocholines in 9 healthy controls and 20 SLE subjects, as determined by the metabolomic scan. Presentation details are as in Fig. 1. The SLE patients have been segregated into 2 groups - mild SLE (SLEDAI <6; N = 10) and active SLE (SLEDAI >5; N = 10). These results are detailed in Supplementary Table S1.
(TIF)

Table S1 **Mean metabolite levels in SLE and healthy control sera.**
(DOCX)
