# Supplementary figures and images for: Metabolic Disturbances Associated with Systemic Lupus Erythematosus
Source: PLoS One. 2012 Jun 19;7(6):e37210. doi: 10.1371/journal.pone.0037210 (PMC3378560; doi:10.1371/journal.pone.0037210)

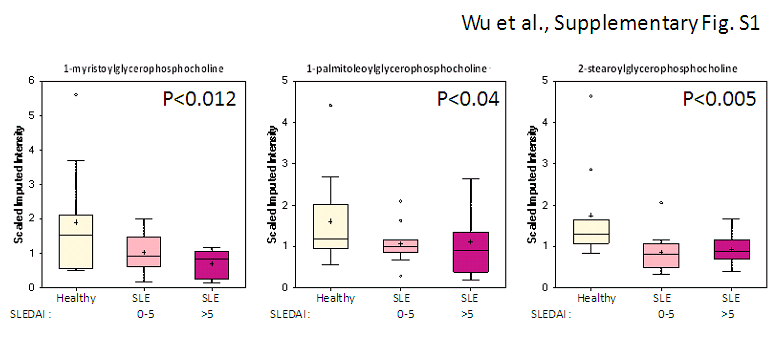

Supplement: Figure S1 — Serum phosphocholine levels in SLE. Plotted are the serum levels of phosphocholines in 9 healthy controls and 20 SLE subjects, as determined by the metabolomic scan. Presentation details are as in Fig. 1. The SLE patients have been segregated into 2 groups - mild SLE (SLEDAI <6; N = 10) and active SLE (SLEDAI >5; N = 10). These results are detailed in Supplementary Table S1. (TIF) [file pone.0037210.s001.tif]
