# Supplementary material for: Metabolic Disturbances Associated with Systemic Lupus Erythematosus
Source: PLoS One. 2012 Jun 19;7(6):e37210. doi: 10.1371/journal.pone.0037210 (PMC3378560; doi:10.1371/journal.pone.0037210)
Supplement: Table S1 — Mean metabolite levels in SLE and healthy control sera. (DOCX) [file pone.0037210.s002.docx]

| **Supplementary Table S1. Mean metabolite levels in SLE and healthy control sera ^1^ .** | | | |  |
| --- | --- | --- | --- | --- |
|  |  |  |  |  |
| **METABOLITE** | **Healthy Control** | **SLE** | **Drug association^2^** |  |
| 1,2-propanediol | 5113916 | 2211199 |  |  |
| 1,3,7-trimethylurate | 72687 | 55977 |  |  |
| 1,5-anhydroglucitol (1,5-AG) | 4543480 | 5697742 |  |  |
| 1,6-anhydroglucose | 282463 | 130138 |  |  |
| 1,7-dimethylurate | 46509 | 36594 |  |  |
| 10-heptadecenoate (17:1n7) | 785015 | 589281 |  |  |
| 10-nonadecenoate (19:1n9) | 338184 | 242536 |  |  |
| 12-HETE + 11-HETE + 8-HETE | 1379096 | 665403 |  |  |
| 13-HODE + 9-HODE | 150902 | 974350 |  |  |
| 1-arachidonoylglycerophosphocholine | 2829180 | 2329711 |  |  |
| 1-arachidonoylglycerophosphoethanolamine | 596454 | 563282 |  |  |
| 1-arachidonoylglycerophosphoinositol | 253999 | 222969 |  |  |
| 1-docosahexaenoylglycerophosphocholine | 959522 | 673163 |  |  |
| 1-eicosadienoylglycerophosphocholine | 138369 | 105506 |  |  |
| 1-eicosatrienoylglycerophosphocholine | 867413 | 743410 |  |  |
| 1-heptadecanoylglycerophosphocholine | 341568 | 247117 |  |  |
| 1-linoleoylglycerol (1-monolinolein) | 350050 | 692496 |  |  |
| 1-linoleoylglycerophosphocholine | 8650601 | 8007940 |  |  |
| 1-linoleoylglycerophosphoethanolamine | 873867 | 874632 |  |  |
| 1-myristoylglycerophosphocholine | 584502 | 256305 |  |  |
| 1-oleoylglycerophosphocholine | 5835978 | 3978042 |  |  |
| 1-oleoylglycerophosphoethanolamine | 1149100 | 1333304 |  |  |
| 1-palmitoleoylglycerophosphocholine | 1203470 | 766521 |  |  |
| 1-palmitoylglycerol (1-monopalmitin) | 65653 | 102845 |  |  |
| 1-palmitoylglycerophosphocholine | 11348027 | 9076107 |  |  |
| 1-palmitoylglycerophosphoethanolamine | 485529 | 679281 |  |  |
| 1-pentadecanoylglycerophosphocholine | 175269 | 131480 |  |  |
| 1-stearoylglycerol (1-monostearin) | 35801 | 45036 |  |  |
| 1-stearoylglycerophosphocholine | 8359443 | 5531392 |  |  |
| 1-stearoylglycerophosphoethanolamine | 925048 | 882634 |  |  |
| 1-stearoylglycerophosphoinositol | 145511 | 254877 |  |  |
| 2-aminobutyrate | 108901 | 92176 |  |  |
| 2-hydroxybutyrate (AHB) | 3255601 | 2521897 |  |  |
| 2-hydroxyglutarate | 83670 | 49088 |  |  |
| 2-hydroxyhippurate (salicylurate) | 296469 | 236214 |  |  |
| 2-hydroxyisobutyrate | 6896094 | 412213 |  |  |
| 2-hydroxyoctanoate | 19264 | 22300 |  |  |
| 2-hydroxypalmitate | 857644 | 822231 |  |  |
| 2-hydroxystearate | 221285 | 200190 |  |  |
| 2-linoleoylglycerophosphocholine | 1199873 | 940933 |  |  |
| 2-linoleoylglycerophosphoethanolamine | 72058 | 67376 |  |  |
| 2-methylbutyroylcarnitine | 47165 | 78648 | Prednisone (0.02) |  |
| 2-oleoylglycerophosphocholine | 362643 | 254351 |  |  |
| 2-oleoylglycerophosphoethanolamine | 60917 | 49655 |  |  |
| 2-palmitoylglycerophosphocholine | 1824189 | 1316191 |  |  |
| 2-palmitoylglycerophosphoethanolamine | 42507 | 45888 |  |  |
| 2-pyrrolidinone | 150238 | 185516 |  |  |
| 2-stearoylglycerophosphocholine | 703684 | 346257 |  |  |
| 3-(4-hydroxyphenyl)lactate | 140268 | 120999 |  |  |
| 3-carboxy-4-methyl-5-propyl-2-furanpropanoate (CMPF) | 253468 | 164574 |  |  |
| 3-dehydrocarnitine | 179571 | 234995 |  |  |
| 3-hydroxybutyrate (BHBA) | 2896497 | 2214046 |  |  |
| 3-hydroxyisobutyrate | 160193 | 183162 |  |  |
| 3-indoxyl sulfate | 2269255 | 2184194 |  |  |
| 3-methoxytyrosine | 22813 | 29150 |  |  |
| 3-methyl-2-oxobutyrate | 213947 | 84903 |  |  |
| 3-methyl-2-oxovalerate | 682438 | 417752 |  |  |
| 3-methylhistidine | 43215 | 53443 |  |  |
| 3-phenylpropionate (hydrocinnamate) | 39418 | 32624 |  |  |
| 4-acetamidobutanoate | 39956 | 50264 |  |  |
| 4-acetaminophen sulfate | 0 | 984381 | Lisinopril (0.02) |  |
| 4-androsten-3beta,17beta-diol disulfate 1 | 281324 | 307727 |  |  |
| 4-androsten-3beta,17beta-diol disulfate 2 | 72957 | 57775 |  |  |
| 4-ethylphenylsulfate | 237392 | 89227 |  |  |
| 4-hydroxyphenylacetate | 39352 | 80858 |  |  |
| 4-methyl-2-oxopentanoate | 1315097 | 612869 |  |  |
| 4-vinylphenol sulfate | 209429 | 122864 |  |  |
| 5,6-dihydrouracil | 48577 | 35779 |  |  |
| 5-acetylamino-6-amino-3-methyluracil | 90309 | 57738 |  |  |
| 5alpha-androstan-3beta,17beta-diol disulfate | 48925 | 87087 |  |  |
| 5alpha-pregnan-3beta,20alpha-diol disulfate | 105066 | 254471 |  |  |
| 5-oxoproline | 1516290 | 733617 |  |  |
| 7-alpha-hydroxy-3-oxo-4-cholestenoate (7-Hoca) | 213592 | 162202 |  |  |
| 7-beta-hydroxycholesterol | 9258 | 15321 |  |  |
| 7-methylguanine | 40956 | 34026 |  |  |
| acetylcarnitine | 1973103 | 1942870 |  |  |
| acetylphosphate | 1223489 | 1251606 |  |  |
| adipate | 100137 | 73120 |  |  |
| ADpSGEGDFXAEGGGVR | 359065 | 1256424 |  |  |
| adrenate (22:4n6) | 820795 | 632089 |  |  |
| ADSGEGDFXAEGGGVR | 210970 | 419892 |  |  |
| alanine | 16431497 | 13993149 |  |  |
| allantoin | 26235 | 19705 |  |  |
| alpha-hydroxyisovalerate | 839080 | 533282 |  |  |
| alpha-ketoglutarate | 40306 | 11395 |  |  |
| alpha-tocopherol | 644164 | 342938 |  |  |
| andro steroid monosulfate 2 | 74130 | 124471 |  |  |
| androsterone sulfate | 425970 | 455197 |  |  |
| arabinose | 60425 | 80492 |  |  |
| arabitol | 41257 | 62457 |  |  |
| arabonate | 39154 | 46861 |  |  |
| arachidonate (20:4n6) | 7611173 | 6058802 |  |  |
| arginine | 628040 | 896106 |  |  |
| asparagine | 226245 | 149635 |  |  |
| aspartate | 978839 | 547679 |  |  |
| aspartylphenylalanine | 503078 | 163892 |  |  |
| azelate (nonanedioate) | 2734371 | 789255 |  |  |
| benzoate | 979847 | 1472534 |  |  |
| beta-alanine | 29973 | 45048 |  |  |
| beta-hydroxyisovalerate | 57438 | 56914 |  |  |
| beta-hydroxypyruvate | 15397 | 32394 |  |  |
| betaine | 860107 | 772837 |  |  |
| bilirubin (E,E) | 211282 | 87253 |  |  |
| bilirubin (Z,Z) | 89093 | 44864 |  |  |
| biliverdin | 196812 | 135458 |  |  |
| bradykinin | 18064 | 49133 |  |  |
| bradykinin, des-arg(9) | 51209 | 864890 |  |  |
| butyrylcarnitine | 115872 | 121296 |  |  |
| caffeine | 2076050 | 867136 |  |  |
| caprate (10:0) | 1622323 | 2069906 |  |  |
| caproate (6:0) | 183361 | 229127 |  |  |
| caprylate (8:0) | 586247 | 726297 |  |  |
| carnitine | 4087390 | 4861722 |  |  |
| catechol sulfate | 640763 | 344045 |  |  |
| C-glycosyltryptophan | 78710 | 115365 |  |  |
| chiro-inositol | 47253 | 40840 |  |  |
| cholate | 29390 | 189939 |  |  |
| cholesterol | 15851091 | 16862099 |  |  |
| choline | 1145577 | 672154 |  |  |
| cis-vaccenate (18:1n7) | 448584 | 333004 |  |  |
| citrate | 2854770 | 2375427 |  |  |
| citrulline | 85454 | 55039 | Prednisone (0.04) |  |
| cortisol | 190094 | 344976 |  |  |
| cortisone | 50926 | 34355 |  |  |
| creatine | 559787 | 592079 |  |  |
| creatinine | 303136 | 411418 |  |  |
| cyclo(leu-pro) | 72841 | 28312 |  |  |
| cysteine | 173026 | 94038 |  |  |
| decanoylcarnitine | 725947 | 252311 |  |  |
| dehydroisoandrosterone sulfate (DHEA-S) | 1378920 | 623943 |  |  |
| deoxycarnitine | 162901 | 143865 |  |  |
| desmethylnaproxen sulfate | 134412 | 0 |  |  |
| dihomo-linoleate (20:2n6) | 1070408 | 761522 |  |  |
| dihomo-linolenate (20:3n3 or n6) | 1454741 | 1125965 |  |  |
| dihydrocholesterol | 46311 | 46707 |  |  |
| dimethylarginine (SDMA + ADMA) | 594187 | 727805 |  |  |
| dimethylglycine | 288882 | 398021 |  |  |
| docosahexaenoate (DHA; 22:6n3) | 2022557 | 1220858 |  |  |
| docosapentaenoate (n3 DPA; 22:5n3) | 599554 | 403190 |  |  |
| DSGEGDFXAEGGGVR | 3488218 | 11030954 |  |  |
| eicosapentaenoate (EPA; 20:5n3) | 698294 | 539227 |  |  |
| eicosenoate (20:1n9 or 11) | 1418923 | 936047 |  |  |
| epiandrosterone sulfate | 209420 | 149368 |  |  |
| ergothioneine | 100695 | 56786 |  |  |
| erythritol | 203115 | 330032 |  |  |
| erythronate | 163534 | 299365 |  |  |
| erythrose | 169408 | 151430 |  |  |
| fructose | 1757828 | 2677297 |  |  |
| fumarate | 65556 | 52577 |  |  |
| gamma-CEHC | 31364 | 29616 |  |  |
| gamma-glutamylalanine | 68248 | 248818 |  |  |
| gamma-glutamylglutamate | 55291 | 248546 |  |  |
| gamma-glutamylglutamine | 181319 | 372673 | Plaquenil (0.05) |  |
| gamma-glutamylisoleucine | 34879 | 202095 |  |  |
| gamma-glutamylleucine | 160396 | 630783 |  |  |
| gamma-glutamylmethionine | 49730 | 142795 |  |  |
| gamma-glutamylphenylalanine | 98201 | 172971 |  |  |
| gamma-glutamylthreonine | 25261 | 71998 |  |  |
| gamma-glutamyltyrosine | 47938 | 79761 |  |  |
| gamma-glutamylvaline | 85589 | 566805 |  |  |
| gamma-tocopherol | 111470 | 118133 |  |  |
| gluconate | 43895 | 291729 |  |  |
| glucose | 78502346 | 105471646 |  |  |
| glucuronate | 126635 | 247688 |  |  |
| glutamate | 310406 | 269120 |  |  |
| glutamine | 507386 | 339999 |  |  |
| glutarate (pentanedioate) | 80857 | 24093 |  |  |
| glutaroyl carnitine | 43115 | 53801 |  |  |
| glycerate | 525884 | 449447 |  |  |
| glycerol | 4662594 | 3444003 |  |  |
| glycerol 2-phosphate | 30069 | 23676 |  |  |
| glycerol 3-phosphate (G3P) | 383642 | 181116 |  |  |
| glycine | 11298696 | 6989613 | Plaquenil (0.03) |  |
| glycochenodeoxycholate | 397503 | 1252888 |  |  |
| glycocholate | 118316 | 320526 |  |  |
| glycocholenate sulfate | 100945 | 148640 |  |  |
| glycodeoxycholate | 161241 | 213708 |  |  |
| glycolate (hydroxyacetate) | 101345 | 108407 |  |  |
| glycolithocholate sulfate | 166959 | 96141 |  |  |
| glycylphenylalanine | 21594 | 23645 |  |  |
| glycylvaline | 192340 | 91293 |  |  |
| guanosine | 67314 | 119566 | Mycophenolic acid (0.04) |  |
| heme | 3887829 | 270112 |  |  |
| heptanoate (7:0) | 208412 | 271444 |  |  |
| hexadecanedioate | 29826 | 46570 |  |  |
| hexanoylcarnitine | 108352 | 85900 |  |  |
| hippurate | 629635 | 503448 |  |  |
| histidine | 367884 | 188197 |  |  |
| HWESASXX | 710760 | 2894180 |  |  |
| HXGXA | 96918 | 93356 |  |  |
| hydroxyisovaleroyl carnitine | 42060 | 40990 |  |  |
| hypoxanthine | 409086 | 89061 |  |  |
| indoleacetate | 220948 | 193759 |  |  |
| indolelactate | 53476 | 52225 |  |  |
| indolepropionate | 109097 | 73518 |  |  |
| inosine | 117081 | 197748 |  |  |
| inositol 1-phosphate (I1P) | 56174 | 47642 |  |  |
| isobutyrylcarnitine | 150983 | 167569 |  |  |
| isoleucine | 9179930 | 7771690 |  |  |
| isovalerate | 62854 | 352972 |  |  |
| isovalerylcarnitine | 98431 | 102511 |  |  |
| kynurenine | 394955 | 484525 |  |  |
| lactate | 273165783 | 119602423 |  |  |
| lathosterol | 22502 | 39270 |  |  |
| laurate (12:0) | 3066741 | 2309956 |  |  |
| laurylcarnitine | 137500 | 62756 |  |  |
| leucine | 16732774 | 11471972 | Prednisone (0.03) |  |
| leucylleucine | 130862 | 420878 |  |  |
| leukotriene B4 | 0 | 464403 |  |  |
| linoleate (18:2n6) | 54666017 | 36610483 |  |  |
| linolenate [alpha or gamma; (18:3n3 or 6)] | 4840925 | 3420927 |  |  |
| lysine | 233271 | 179748 |  |  |
| malate | 136856 | 47800 |  |  |
| mannitol | 132671 | 244008 |  |  |
| mannose | 1856098 | 2865024 |  |  |
| margarate (17:0) | 1714348 | 1327729 | Cytoxan (0.006) |  |
| meprobamate | 602124 | 0 |  |  |
| methionine | 1678708 | 1286224 |  |  |
| methyl palmitate | 77732 | 66041 |  |  |
| methylglutaroylcarnitine | 32255 | 78629 |  |  |
| methylphosphate | 221263 | 179566 |  |  |
| myo-inositol | 1850839 | 2102440 |  |  |
| myristate (14:0) | 6284270 | 4412215 |  |  |
| myristoleate (14:1n5) | 492868 | 324216 |  |  |
| N-(2-furoyl)glycine | 50559 | 55100 |  |  |
| N1-methyladenosine | 81123 | 88500 |  |  |
| N2,N2-dimethylguanosine | 27000 | 60720 |  |  |
| N4-acetylcytidine | 22451 | 42328 |  |  |
| N6-acetyllysine | 85055 | 61854 |  |  |
| N6-carbamoylthreonyladenosine | 26284 | 52464 |  |  |
| N-acetylalanine | 27714 | 28808 |  |  |
| N-acetylglycine | 122366 | 119325 |  |  |
| N-acetylneuraminate | 54632 | 64291 |  |  |
| N-acetylornithine | 69486 | 91846 |  |  |
| N-acetylserine | 27075 | 43376 |  |  |
| n-Butyl Oleate | 36203 | 44039 |  |  |
| N-methyl proline | 115191 | 56881 |  |  |
| nonadecanoate (19:0) | 97756 | 81552 |  |  |
| octadecanedioate | 60989 | 68227 |  |  |
| octanoylcarnitine | 439831 | 162652 |  |  |
| oleate (18:1n9) | 2728835 | 1689732 |  |  |
| oleoylcarnitine | 561686 | 277329 |  |  |
| ornithine | 653979 | 162291 |  |  |
| palmitate (16:0) | 55298030 | 38414811 |  |  |
| palmitoleate (16:1n7) | 9285873 | 6212856 |  |  |
| palmitoyl sphingomyelin | 8089011 | 8383857 |  |  |
| pantothenate | 140477 | 80235 |  |  |
| paraxanthine | 418943 | 202620 |  |  |
| p-acetamidophenylglucuronide | 0 | 667144 | Lisinopril (0.04) |  |
| p-cresol sulfate | 4914602 | 3082467 |  |  |
| pelargonate (9:0) | 4067799 | 5623363 |  |  |
| pentadecanoate (15:0) | 94131 | 72736 |  |  |
| phenol sulfate | 1203633 | 1177624 |  |  |
| phenylacetate | 27300 | 36211 |  |  |
| phenylacetylglutamine | 552484 | 475007 |  |  |
| phenylalanine | 20462142 | 14730443 | Prednisone (0.007) |  |
| phosphate | 65960344 | 33191748 |  |  |
| pipecolate | 270101 | 241420 |  |  |
| piperine | 508592 | 686328 |  |  |
| pregn steroid monosulfate | 215591 | 134296 |  |  |
| pregnen-diol disulfate | 168705 | 182559 |  |  |
| pro-hydroxy-pro | 184748 | 261361 |  |  |
| proline | 3737362 | 3504922 |  |  |
| propionylcarnitine | 304434 | 332284 |  |  |
| pseudouridine | 101235 | 174263 |  |  |
| pyridoxal | 119919 | 40198 |  |  |
| pyridoxate | 785941 | 67177 |  |  |
| pyroglutamine | 37497 | 88482 |  |  |
| pyroglutamylglycine | 136528 | 82204 |  |  |
| pyroglutamylvaline | 0 | 67434 |  |  |
| pyrophosphate (PPi) | 238893 | 246948 |  |  |
| pyruvate | 1250787 | 173660 |  |  |
| quinate | 329785 | 91021 |  |  |
| riboflavin (Vitamin B2) | 56770 | 25138 |  |  |
| ribose | 161127 | 69051 |  |  |
| saccharin | 0 | 107369 |  |  |
| salicylate | 160809 | 425341 |  |  |
| scyllo-inositol | 53175 | 80434 |  |  |
| sebacate (decanedioate) | 442927 | 89443 |  |  |
| serine | 2366822 | 1855537 |  |  |
| serotonin (5HT) | 140108 | 102834 |  |  |
| sorbitol | 18353 | 26541 |  |  |
| stachydrine | 993174 | 1345331 |  |  |
| stearate (18:0) | 31053412 | 22885924 |  |  |
| stearidonate (18:4n3) | 56356 | 56556 |  |  |
| stearoyl sphingomyelin | 854778 | 1040320 |  |  |
| stearoylcarnitine | 83726 | 62665 |  |  |
| suberate (octanedioate) | 136701 | 58445 |  |  |
| succinate | 52002 | 48620 |  |  |
| succinylcarnitine | 38828 | 36584 |  |  |
| tartronate (hydroxymalonate) | 172608 | 162735 |  |  |
| taurochenodeoxycholate | 102160 | 299109 |  |  |
| taurocholate | 44554 | 135739 |  |  |
| taurocholenate sulfate | 74009 | 60283 |  |  |
| tetradecanedioate | 21337 | 27453 |  |  |
| theobromine | 757219 | 233311 |  |  |
| theophylline | 103608 | 69175 | Mycophenolic acid (0.03) |  |
| threitol | 71422 | 96998 |  |  |
| threonate | 423647 | 207529 |  |  |
| threonine | 133442 | 97579 |  |  |
| threonylphenylalanine | 29338 | 56835 |  |  |
| thymol sulfate | 109655 | 210563 |  |  |
| trans-4-hydroxyproline | 31705 | 37765 |  |  |
| tryptophan | 14291577 | 11152723 | Prednisone (0.05) |  |
| tryptophan betaine | 654594 | 278336 | Mycophenolic acid (0.005) |  |
| tyrosine | 8397271 | 7188575 |  |  |
| undecanedioate | 726792 | 136233 |  |  |
| undecanoate (11:0) | 329490 | 367877 |  |  |
| uracil | 58989 | 8652 |  |  |
| urate | 4983058 | 8104775 | Prednisone (0.001) |  |
| urea | 49156248 | 71073188 |  |  |
| uridine | 504418 | 298058 | Cytoxan (0.007) |  |
| urocanate | 28891 | 28557 |  |  |
| valerate | 35211 | 25072 |  |  |
| valine | 13164582 | 9983718 | Prednisone (0.001) |  |
| vanillin | 59590 | 51137 |  |  |
| xanthine | 66324 | 32401 |  |  |
| xylitol | 9229 | 10503 |  |  |
| xylose | 136923 | 85921 |  |  |
|  |  |  |  |  |
| 1: Plotted are the mean raw values of the different serum metabolites in the SLE patients (N=20) | | | |  |
| and healthy controls (N=9). These values were normalized and subjected to statistical analysis | | | |  |
| to identify metabolites that were significantly different in SLE. Listed are all named metabolites | | | |  |
| that were identifiable when compared to a library of >2000 metabolites. Of these metabolites, | | | |  |
| 89 were significantly different in patients with mild SLE (SLEDAI<6) compared to healthy controls, | | | |  |
| while 88 metabolites were significantly different in patients with active disease (SLEDAI>5) | | | |  |
| compared to controls. Finally, an additional 40 metabolites were different in SLE sera at a | | | |  |
| statistical significance p-value of 0.05-0.10, compared to healthy controls. | | |  |  |
|  |  |  |  |  |
| 2: Indicated are metabolites that were significantly associated with the patients | | |  |  |
| on particular medications. Indicated in paranthesis is the P value. | |  |  |  |
